# Aging retinal pigmented epithelium: omics-based insights into vision decline

**DOI:** 10.18632/aging.205914

**Published:** 2024-06-12

**Authors:** Ioan V. Matei, Luminita Paraoan

**Affiliations:** 1 Faculty of Arts and Sciences, Edge Hill University, Ormskirk, Lancashire L39 4QP, UK

**Keywords:** RPE, vision, aging, omics

Of all senses affected by aging, vision decline arguably has the most impactful relationship with overall wellbeing, health and personal autonomy. However, while the ensuing importance of vision loss has long been recognised from a public health perspective given an increasingly aging population, understanding the molecular and cellular mechanisms driving age-related pathological changes is still in its infancy. This matter is, therefore, critical for tackling sensory impairment and ensuring healthy aging.

The retinal pigmented epithelium (RPE), the cellular monolayer located between the neuroretina and the highly vascularized choroid, from which it is separated by Bruch’s membrane (BrM), has a critical role in human vision and performs essential functions throughout life for maintaining the retinal homeostasis. RPE is a specialised, fully differentiated tissue that is mitotically inactive, with no regenerative potential. Unsurprisingly, given all its characteristics, functions and metabolic demands, the RPE is particularly susceptible to aging, sustaining significant morphologic and physiologic changes. Aging is recognised as the highest risk factor for age-related macular degeneration (AMD), the leading cause of adult visual impairment and blindness in the Northern Hemisphere, which is directly linked to specific pathological changes of the RPE located in the macula, i.e., the central part of retina; these changes, therefore, affect central vision required for reading, driving, and discerning details of pictures, faces, shapes and colors.

Molecular omics profiling of the aging RPE has been slower in contrast to other tissues, owing mostly to the scarcity and anatomical difficulties of accessing normal aging tissue. Normal RPE gene/protein expression datasets have been initially acquired as age-matched RPE controls in relation to and for comparison with the AMD RPE, thus presenting limited scope for age range and for subsequent analysis of molecular mechanism of specific age-related core RPE functions.

Recent transcriptomic studies purposely focusing on characterizing age-related changes in the human RPE are providing new insights into the complex regulation of retinal processes, such as the surprising age-related increase in visual cycle gene transcription, notably *LRAT, RPE65, RDH5, RDH10, RDH11* [1] Consequent functional investigation found that *LRAT* expression was positively upregulated by the presence of visual cycle condensation by-products, such as bis-retinoid-pyridinium-ethanolamine (A2E), - a constituent of lipofuscin, associated with both the aging RPE and the emergence of AMD - and all-*trans*-retinal dimer, resulting from a failure in the reduction of all-*trans*-retinal (atRAL) to retinol [1]. Similar disturbances in the visual cycle have also been reported for Stargardt disease, ABCA4 retinopathies and dry AMD.

Earlier studies described increased RPE topographic/regional heterogeneity with regard to cell distribution and density with aging. For example, the ability of peripheral RPE cells to migrate in order to compensate apoptotic age-related loss of cells in the central retina was noted in human studies [2], showing an age-related increase in apoptotic RPE cells coupled with constant cell density in the macula and reduced cell density in the periphery.

Single cell RNAseq (scRNAseq) studies provided potential functional links for the regional RPE heterogeneity [3], identifying two major RPE cell subpopulations: an *ID3*-expressing macular cluster and an *CRYAB*-expressing peripheral cluster, with the former exhibiting higher expression of genes involved in protection against oxidative and ER stress, and the latter presenting higher expression of genes involved in light perception, oxidative stress and lipid metabolism [4]. Another scRNAseq analysis of the RPE and choroid surveying cca. 300,000 qualified cells across both regions revealed that RPE cells constitute about 1.5% of the surveyed cells in the periphery and about 2.5% of the central region, averaging about 2% overall, and showing a steep decrease with age to 0.8% at 56 years, and approx. 0.25% at 81 years, concurrently with a topographically uneven gene expression pattern [5]. Aging resulted in a down-regulation of extracellular matrix genes such as *ELN* and *COL1A1* and endocytosis-related genes such as *IST1* and *WASHC1* in RPE cells as well as other surveyed cell populations [5]. Of note, ECM/BrM dysfunction has also been linked to AMD emergence.

The accumulation of somatic mutations, as ascertained through single nucleotide variants (SNVs) and copy number variations (CNVs), tends to be much higher in the central RPE, likely due to the high metabolic stress and elevated temperature of this particular niche. By contrast, the peripheral RPE and choroid show fairly similar rates of somatic mutation [5]. Overall, the somatic mutation accumulation rate was shown to increase linearly with age [5]. The topographical heterogeneity was further reflected by another scRNAseq study revealing that central loss of RPE cells and melanocytes can be detected in intermediate stages of AMD [6].

Senescent RPE was linked to the onset of AMD, with senolytic agents assessed as potential treatments for AMD [7]. A fact that has been seldom considered among the plethora of processes that are affected and, in turn, effected by cellular senescence (an adaptive cell response to stressors effected by an inflammatory secretome) is that of intercellular communication. In the case of the RPE, its functional and anatomical ties to the choroid are as much, if not more so, functionally relevant as those with the neuroretina. A study applying a combined approach using bulk RNAseq, scRNAseq and microarray RPE transcriptomics was able to draw substantial inferential data pertaining to intercellular communication between the RPE and the choroid [8]. Intercellular communications between RPE cells and stromal elements involved in aging and senescence pertained notably to VEGF, BMP-and tenascin-mediated pathways, i.e., these pathways scored higher when mediating interactions between senescent cell populations [8]. Consistently, AMD-derived patient samples scored higher overall in terms of senescence, and bulk RNAseq data showed a positive correlation in score increase with age [8]. These findings potentially support employing anti-aging therapies such as senolytic pharmacologic compounds to prevent or ameliorate progression to AMD, as well as underscore the necessity of more rigorous investigation into the interplay of senescence and cell-to-cell communication.

In recent years, a wide array of omics-based approaches has been deployed to provide a deeper understanding of the mechanistic basis of the substantial morphologic and functional decline exhibited by the aged RPE. Similarities were highlighted between age-related changes in RPE physiology and changes induced in RPE cells by AMD-associated gene variants [9] and other macular disorders. Whether the pathological changes and impaired cellular processes affected by aging are the same as those due to risk variants – and therefore accelerated in aging – is still an open scientific question, and one of great fundamental and translational relevance. Comprehensive and interventional *in vitro* studies into the modifications, both structural and functional, underpinning age-related RPE decline could shed light on the key mechanisms by virtue of which these disruptions occur, thus yielding profound and far-reaching outcomes for both AMD fundamental knowledge and translational approaches.
